# Sp1 Is Essential for p16^INK4a^ Expression in Human Diploid Fibroblasts during Senescence

**DOI:** 10.1371/journal.pone.0000164

**Published:** 2007-01-17

**Authors:** Junfeng Wu, Lixiang Xue, Mo Weng, Ying Sun, Zongyu Zhang, Wengong Wang, Tanjun Tong

**Affiliations:** Peking University Research Center on Aging, Department of Biochemistry and Molecular Biology, Peking University, Health Science Center, Beijing, China; University of Munich, Germany

## Abstract

**Background:**

*p16* ^INK4a^ tumor suppressor protein has been widely proposed to mediate entrance of the cells into the senescent stage. Promoter of *p16* ^INK4a^ gene contains at least five putative GC boxes, named GC-I to V, respectively. Our previous data showed that a potential Sp1 binding site, within the promoter region from −466 to −451, acts as a positive transcription regulatory element. These results led us to examine how Sp1 and/or Sp3 act on these GC boxes during aging in cultured human diploid fibroblasts.

**Methodology/Principal Findings:**

Mutagenesis studies revealed that GC-I, II and IV, especially GC-II, are essential for *p16* ^INK4a^ gene expression in senescent cells. Electrophoretic mobility shift assays (EMSA) and ChIP assays demonstrated that both Sp1 and Sp3 bind to these elements and the binding activity is enhanced in senescent cells. Ectopic overexpression of Sp1, but not Sp3, induced the transcription of *p16* ^INK4a^. Both Sp1 RNAi and Mithramycin, a DNA intercalating agent that interferes with Sp1 and Sp3 binding activities, reduced *p16* ^INK4a^ gene expression. In addition, the enhanced binding of Sp1 to *p16* ^INK4a^ promoter during cellular senescence appeared to be the result of increased Sp1 binding affinity, not an alteration in Sp1 protein level.

**Conclusions/Significance:**

All these results suggest that GC- II is the key site for Sp1 binding and increase of Sp1 binding activity rather than protein levels contributes to the induction of *p16* ^INK4a^ expression during cell aging.

## Introduction

Normal human cells undergo a finite number of cell divisions and ultimately enter a nondividing state called replicative senescence[1]. Replicative or cellular senescence was observed and proposed as an experimental model for aging at the cellular level over thirty years ago. Senescent cells remain metabolically active and show characteristic changes in cell morphology, physiology and gene expression [2]–[4]. The mechanism of aging is very complex and it implicates genetics, environment, psychology and sociology, while the genetic factor is critical for life span. There are several independent pathways that control the process of replicative senescence in human cells [5]–[9]. Such pathways often involve the activation of the cell cycle inhibitors, *p21* ^CIP1/WAF1^ [10]–[12] and *p16* ^INK4a^ [13]–[18], which are likely the genes that act to induce cellular senescence, and are in fact direct targets of the genetic program that leads cells to senescence [19], [20].

It is well known that p16^INK4a^ inhibits cdk (cyclin-dependent kinase)4/cdk6-mediated phosphorylation of retinoblastoma gene product (pRb) and induces cell cycle arrest in G1 phase [21], *p16* ^INK4a^ up-regulation is also a key event in the terminal stages of growth arrest in senescence. The mechanism of increased *p16* ^INK4a^ expression in senescent cells is not well understood. We and others previously reported that some regulatory factors, such as Bmi-1, Ets, Id1, E47, Jun B, p21^CIP1/WAF1^and ITSE binding factor, were involved in *p16* ^INK4a^ transcription[22]–[27]. Here we show that transcription factor Sp1 also plays an important role in the regulation of *p16^INK4a^* gene expression during senescence. The increase of Sp1 binding activity rather than the protein level in senescent fibroblasts contributes to the high levels of p16^INK4a^ expression during the progress of aging. What's more, the basal level of Sp1 is essential for this effect.

## Materials and Methods

### Cell culture

Human embryonic lung fibroblast cell line (2BS cells) obtained from the National Institute of Biological Products (Beijing, China), was cultured in DMEM medium, containing 10% fetal bovine serum, 100 U/ml penicillin and 1 µg/ml streptomycin[28], [29].

### Plasmids construction

To make pGL3-620, 620bp 5′-fragment of human *p16INK4a* promoter was digested with Rsa I and Hind III from pSIR-870-EGFP, and inserted into pGL3-Basic Luciferase Report Vector (Promega). Site-directed mutagenesis was conducted using Quickchange™ Site-directed mutagenesis Kit (Stratagene) according to the manufacturer's protocol. Five oligo nucleotides carrying mismatched bases: 5′-GGAAGGAAACGGatCcGGGGCGGATTTC-3′, 5′-GGCGGGGGCaGATcTCTTTTTAACAGAG-3′, 5′-GGGAGGCCGGAtccCGGTGTGGGGGG-3′, 5′-CAGAGGGTGGGGgGatCCGAGTGCGCTC-3′ and 5′-GCAGGCAGCGGGatcCGGGGAGCAGC-3′ were used to mutagenize the segments containing GC boxes spanning from nt (nucleotide) −474 to −447, −462 to −435, −380 to −355, −76 to −49 and −26 to −1 upstream of translational start site respectively (Table 1). The nucleotide sequences of the mutants were confirmed by sequencing. Expression plasmids of Sp1 and Sp3 were kindly provided by Dr. Robert Tjian (University of California, Berkeley, CA) and Dr. G. Suske (IMT, Marburg, Germany) respectively. The pCMV plasmid without an insert served as the control.

**Table 1:**
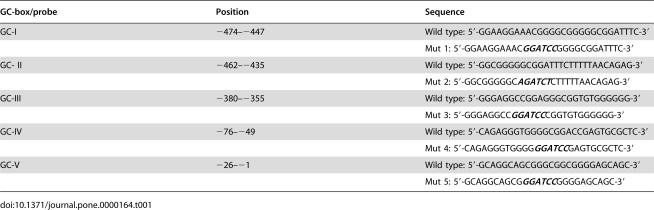
Serial mutagenesis of GC-box in the 5′-flanking regions of the human *p16^INK4a^*.

GC-box/probe	Position	Sequence
GC-I	−474–−447	Wild type: 5′-GGAAGGAAACGGGGCGGGGGCGGATTTC-3′
		Mut 1: 5′-GGAAGGAAAC***GGATCC***GGGGCGGATTTC-3′
GC- II	−462–−435	Wild type: 5′-GGCGGGGGCGGATTTCTTTTTAACAGAG-3′
		Mut 2: 5′-GGCGGGGGC***AGATCT***CTTTTTAACAGAG-3′
GC-III	−380–−355	Wild type: 5′-GGGAGGCCGGAGGGCGGTGTGGGGGG-3′
		Mut 3: 5′-GGGAGGCC***GGATCC***CGGTGTGGGGGG-3′
GC-IV	−76–−49	Wild type: 5′-CAGAGGGTGGGGCGGACCGAGTGCGCTC-3′
		Mut 4: 5′-CAGAGGGTGGGG***GGATCC***GAGTGCGCTC-3′
GC-V	−26–−1	Wild type: 5′-GCAGGCAGCGGGCGGCGGGGAGCAGC-3′
		Mut 5: 5′-GCAGGCAGCG***GGATCC***GGGGAGCAGC-3′

### Transfection and luciferase activity assays

All plasmids were purified with Qiagen Plasmid Midi Kits (Chatsworth, CA). For each transfection experiment, 2BS cells were seeded at 12-well plates and grown for about 24 h until they were above 90% confluent, then transfected with an equal amount of reporter plasmid (1.6 µg) and 0.32 µg of pRL-CMV (Promega) as transfection efficiency control, using lipofectamine 2000 (Invitrogen) and following the manufacturer's indications. Five hours later, serum-free DNA-containing medium was replaced by fresh growth medium and the cells were harvested 48h after transfection. Luciferase assays were performed as described (Dual-Luciferase Reporter Assay System, Promega). All assays were carried out in triplicate and performed twice for confirmation.

### RNA analysis

Total RNA was prepared from exponentially growing cells using RNeasy Mini Kit (Qiagen, Hilden, Germany). For Northern blot analysis, RNA was electrophoresed in 1.5% formaldehyde-denaturing agarose gel, with the 0.24–9.5 kb RNA Ladder included in one lane as a size marker (Gibco BRL, Gaithersburg, MD). RNA was then transferred onto Biodyne B membrane (Pall, East Hills, NY) according to manufacturer's recommendations and fixed. The human p16^INK4a^ gene-coding region was used as probe labeled with [α-^32^P] dCTP (Yahui, Beijing, China) by random priming using the Prime-a-Gene Labeling System (Promega). Hybridization was carried out in ExpressHyb™ Hybridization Solution (Clontech) at 68°C for 1 h. The blot was stringently washed with 0.1×SSC, 0.1% SDS at 68°C for 15 min twice. Autoradiography was performed at −80°C. The same blot was stripped after probing and then reprobed with the ^32^P-labeled GAPDH probe to control for equivalent RNA loading in each lane.

### Western blot

Cell extracts were prepared following standard procedures. Briefly, three to five volumes of lysis buffer (50 mM Tris-HCL, pH 7.4, 0.25 M NaCl, 0.1% Triton-X-100, 1 mM EDTA, 50 mM NaF, 1 mM DTT, 0.1 mM Na_3_VO_4_) were added to a cell pellet. The following protease inhibitors were added: 0.1 mM PMSF, 1 µg/ml leupeptin, 1 µg/ml aprotinin). After incubation on ice for 30 min, samples were centrifuged at 14,000 r.p.m. for 5 min at 4°C to recover the supernatant. After proteins were electrophoresed in a 15% (for p16) or 8% (for Sp1/Sp3) denaturing polyacylamide gel and transferred to a PVDF membrane, the membrane was blocked in 5% nonfat milk-TBS-0.25% Tween 20 for 1 h and incubated with the primary antibody in TBS-0.25% Tween 20 for 1 h at room temperature. Complexes were detected with horseradish peroxidase-linked secondary antibody and enhanced chemiluminescence (SuperSignal, Pierce). The primary and secondary antibodies used in this study were all from Santa Cruz Biotechnology (Santa Cruz).

### Preparation of nuclear extract*s*

Nuclear extracts from 2BS cells were prepared as follows: About 1×10^6^ cells were harvested, washed twice with cold PBS (phosphate-buffered saline) and collected by centrifugation at 3500 rpm at 4°C for 5 min. The cells were resuspended in 400 µl of solution I (10 mM HEPES, pH 7.9, 10 mM KCl, 1.5 mM MgCl_2_, 0.1 mM EDTA, 0.1 mM DTT, 0.5 mM PMSF) and lysed by passing them through a 25 gauge syringe. Nuclei were pelleted at 2,000 r.p.m for 10 min, washed once with solution I and resuspended in 200 µl of solution II (solution I with 5% glycerol, 400 mM NaCl and without KCl). The suspension was rotated at 4°C for 30 min and then centrifuged at 14,000 r.p.m, 4°C, for 30 min. The resulting clear supernatant was stored at −80°C until use.

### Electrophoretic mobility shift Assay (EMSA) and supershift assay

A 38 bp long oligonucleotide 5′-GGAAGGAAACGGGGCGGGGGCGGATTTCTTTTTAACAG-3′ (oligo I) extending from nt −474 to −437 or a 28 bp long oligonucleotide 5′-CAGAGGGTGGGGCGGACCGAGT GCGCTC-3′ (oligo II) extending from nt −76 to −49 on the 5′ UTR of p16 gene, was used in EMSA and supershift assay. Double stranded DNA fragments were end-labeled with [γ-^32^P] dATP (Yahui, Beijing, China) and T4 polynucleotide kinase (New England Biolabs, Beverly, MA). The probes were purified using QIAquick Nucleotide Removal Kit (Qiagen) and 15,000 cpm were incubated for 25 min on ice with 10 µg of nuclear extract from 2BS cell line, in the presence of 20 mM Tris-HCl pH 7.5, 75 mM KCl, 3.5 mM DTT, 20 nM ZnCl_2_, 1 µg/µl BSA, 5% glycerol and 1 µg poly (dI-dC) (Pharmacia Biotech, Piscataway, NJ) in a total volume of 20 µl. For supershift experiments, 2 µg of antibody against Sp1 or Sp3 (Santa Cruz Biotechnology, Santa Cruz, CA) was incubated for 20 min on ice with the nuclear extract before adding the labeled probe. DNA/protein complexes were separated from free DNA on a 5% polyacrylamide gel in TBE (89 mM Tris, 89 mM boric acid, 2 mM EDTA) at 4°C for 120 min at 340 V. After electrophoresis, gels were dried and autoradiographed.

### Chromatin immunoprecipitation (ChIP)

ChIPs were performed using the Chromatin Immunoprecipitation Assay Kit (Upstate, New York) according to manufacturer's instruction. In brief, 1×10^6^ cells were crosslinked by adding formaldehyde directly to cell culture media and incubated for 10 min at 37°C. Wash cells twice with cold PBS and then cells were scraped and resuspended in 200 µl of SDS Lysis Buffer. Chromatin was then sonicated to an average length of 0.5 Kb for three 30 sec. pulses at maximum power. Chromatin extracts were diluted 10 folds in Dilution Buffer and preincubated for 30 min at 4°C with 80 µl of Salmon Sperm DNA/ protein A Agarose. Twenty microlitres of diluted supernatant was kept for isolation of input DNA and to quantitate the DNA in different samples. After pelleting agarose by brief centrifugation, 2 µg of anti-Sp1 antiserum (test group) or 2 µg of β-actin antibody (irrelevant antibody control) was added to the supernatant fraction and incubated overnight at 4°C with rotation. In addition, perform a no antibody immunoprecipitation by incubating the supernatant fraction with Salmon Sperm DNA/ protein A Agarose for 1 h at 4°C. Add 60 µl of Salmon Sperm DNA/ protein A Agarose for 1 h at 4°C to collect the antibody/antigen-DNA complex. The chromatin bound to the protein A Agarose beads was eluted in 500 µl of freshly prepared elution buffer (1% SDS, 0.1 M NaHCO_3_). After reversing crosslinks, the samples were deproteinized and phenol-chloroform extracted, then DNA was ethanol precipitated using yeast tRNA as a carrier. Pellets were resuspended in 50 µl of TE buffer for PCR analysis.

Each PCR reaction mixture contained 5 µl of immunoprecipitated chromatin in a final reaction volume of 20 µl. PCR mixtures were amplified for 35 cycles of 94°C for 30 s, 54°C for 30 s, and 72°C for 30 s. To amplify GC-box containing regions of *p16^INK4a^* promoter, the sequences of the primers used were as follows: ChIP1, 5′-CCCCGATTCAATTTGGCAGTTAGG-3′ (−505–−491); ChIP2, 5′-CAGCGTTGGCAAGGAAGGAG GAC-3′ (−342–−318). All primers were synthesized at AuGCT Biotechnology Co., Ltd (Beijing, China).

### RNA interference(RNAi)

The target sequence against mRNA of Sp1 is 5′-AUCACUCCAUGGAUGAAAUGATT-3′, which has been reported to be effective in some cell lines [30]. The hairpin siRNA(small interference RNA) template oligonucleotides containing the target sequence is designed following pSilencer neo instruction manual (Ambion, USA), and was chemically synthesized, annealed and inserted into the BamH I and Hind III site of pSilencer U6 2.1 neo vector (Ambion, USA).

Cells were transfected using Lipofectamin 2000 as specified by the manufacturer. The transfection mixture was left on the cells for 4 h, after which DMEM/20% serum without antibiotics was added. For efficient knockdown two more transfections were performed at 24 h and 48 h after the first transfection.

### SA-β-Galactosidase activity at pH 6.0

Cells were washed twice in PBS, fixed in 3% formaldehyde, and washed again in PBS. The cells were incubated overnight at 37°C (without CO_2_) with freshly prepared SA-β-Gal staining solution.

## Results

### GC boxes are essential for *p16^INK4a^* promoter activity in senescent human fibroblast cells

**Figure 1:**
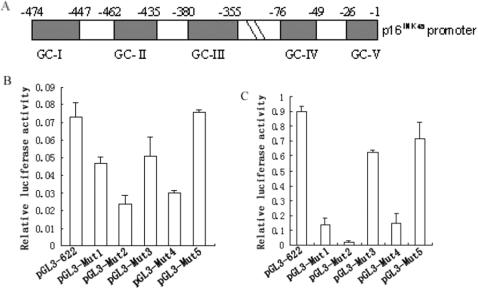
p16^INK4a^ transcription activity increased with cell senescence. A. Schematic presentation of mutants of GC-boxes in the *p16^INK4a^* promoter. Young (B) and senescent (C) 2BS cells were transfected with 1.6 µg GC-box mutants or pGL3-620 along with 0.32 µg pRL-CMV vectors. 48 hours after transfection, cells were harvested and subjected to luciferase activity assays. The luciferase activity was normalized to Renilla luciferase activity. The data represent the mean.±S.E. of three independent experiments.

To determine the crucial GC-rich region of the human *p16^INK4a^* promoter, plasmid pGL3-620 that contained the *p16^INK4a^* promoter with 620 bp upstream of the translation start site was used to generate GC boxes site mutation constructs pGL-Mut 1∼5 (Tab. 1, and Fig. 1A) respectively. These plasmids were then individually transfected into young (Fig. 1B) or senescent (Fig. 1C) human embryonic lung fibroblasts 2BS cells, and the promoter activities were measured by luciferase activities. Results showed that both in young and senescent cells, the mutation of GC-I, II or GC-IV reduced the promoter activity, while the mutation of GC-III or GC-V had less significant effect. What'more, the effect is stronger in the senescent cells than in the young ones. Therefore, GC-I, II and GC-IV, especially GC-II, are the key sites of action for Sp1 induced *p16^INK4a^* expression. Furthermore, compared the relative value of these groups, it appears that the promoter activity of the human *p16^INK4a^* is much more potent in senescent cells than in young ones.

### Senescent cells contain potent Sp1/Sp3 binding activities to the GC boxes of the *p16^INK4a^* promoter

**Figure 2:**
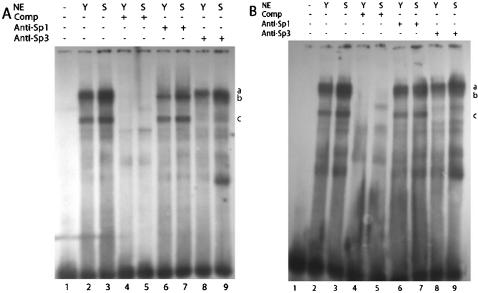
Binding of Sp1 and Sp3 to the *p16^INK4a^* promotor. Electrophoretic mobility shift assays (EMSA) was performed using nuclear extracts (NE) from young (Y) or senescent (S) 2BS cells and radiolabeled oligo I (A) or oligo II (B). Competition was performed in the presence of 100-fold molar excess of the cold synthetic oligos (Comp) as indicated. The major specific complexes are indicated as a, b and c. The presence of Sp1 and Sp3 in the DNA-protein complexes was monitored by the disappearance of the retarded bands in the presence of antibodies against Sp1 and Sp3 (supershift).

To evaluate whether Sp proteins indeed binds to the GC boxes, gel electrophoretic mobility shift assays (EMSA) were performed. Oligo I including GC-I and GC-II and oligo II containing GC-IV were 5′end labeled with [γ-^32^P-]dATP and incubated with nuclear extracts from either young or senescent 2BS cells. The DNA-protein binding complexes were then analyzed on 5% polyacrylamide gel. As shown in Figure 2A, three specific DNA-protein binding complexes a, b and c, were detected by using oligo I, and all of the complexes disappeared with the addition of 100-fold molar excess of unlabeled competitor. To confirm the presence of Sp proteins in these complexes, Sp1 monoclonal antibodies or Sp3 polyclonal antibodies were incubated with the nuclear extracts before the DNA-protein binding complexes being analyzed on the gel. As shown in Figure 2A, complex a disappeared with the addition of Sp1-specific antibody; while complex b and c disappeared by using Sp3-specific antibody. Similar results were obtained with radio-labeled oligo II as probes, as demonstrated in Figure 2B. What is also worth noting is that Sp1/Sp3 DNA binding affinity in senescent cells is about 78% higher than that in young ones. This result is consistent with our earlier observation (Fig. 1) that the *p16^INK4a^* promoter had much potent activity in senescent cells than that of young ones.

### Sp1/Sp3 binds to *p16^INK4a^* promoter in vivo

**Figure 3:**
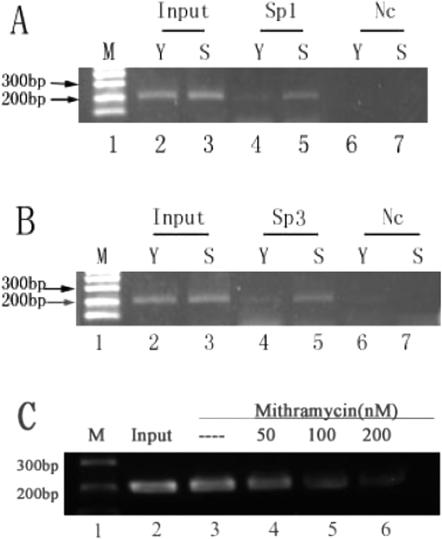
Sp1/Sp3 bound to the GC boxes containing region in the *p16^INK4a^* gene promoter in 2BS cells. A and B. ChIPs assays of young (Y: PD27) and senescent (S: PD60) 2BS cells using antibodies against Sp1 (A) or Sp3 (B), antibody against β-actin was used as irrelevant control (Nc). (C) Senescent (S: PD60) 2BS cells were treated with MTR in the dosage indicated, 24 hours later, cells were harvested and subjected to ChIP assays. Data are representative of three independent experiments.

We next want to know whether Sp1 and/or Sp3 could bind to *p16^INK4a^* promoter in living 2BS cells. For this purpose, we performed ChIP assay to monitor Sp1- and Sp3-occupancies in the *p16^INK4a^* promoter using gene specific primers. Consistent with the results of EMSA, ChIP assays showed that Sp1/Sp3 indeed bound to *p16^INK4a^* promoter in vivo and the binding activity in senescent cells was higher than in that of young cells by nearly 5 folds (Sp1) and 3.5 folds (Sp3) respectively (Fig. 3A, Fig. 3B).

### Sp1 induces *p16^INK4a^* promoter activation and both Sp1 binding and basal level of Sp1 are critical to the expression of *p16^INK4a^*

**Figure 4:**
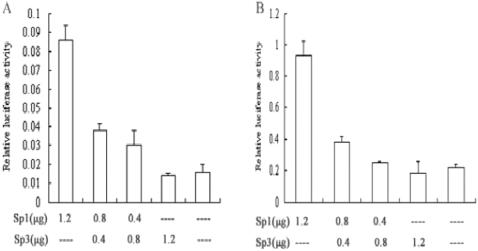
Effect of Sp1 and Sp3 on *p16^INK4a^* promoter activity. The reporter construct pGL3-620 was co-transfected with pCMV-Sp1 or/and pCMV-Sp3 or control vector along with pRL-CMV in young (A) and senescent (B) 2BS cells. Luciferase assays were performed and normalized to the Renilla luciferase activity. The mean±S.E. from three independent experiments was used to express the relative luciferase activity.

To analyze the effect of Sp1 and Sp3 on *p16^INK4a^* promoter, Sp1 and/or Sp3 expression vectors were co-transfected with pGL3-620 into young or senescent 2BS cells. As determined by luciferase activity, Sp1 activated the *p16^INK4a^* promoter in a dose-dependent manner in both young and senescent 2BS cells, while Sp3 had little effect on it (Fig. 4).

**Figure 5:**
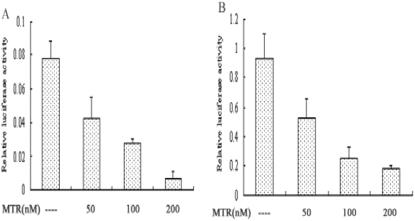
Effect of MTR on the transcriptional activity of the *p16^INK4a^* gene promoter. Young (A) and senescent (B) 2BS cells were transfected with pGL3-620. 24 hours after transfection, cells were exposed to different dosage of MTR (M-A) as indicated for 24 more hours and subjected to luciferase activity assays. The data represent the means±S.E. of five independent experiments.

**Figure 6:**
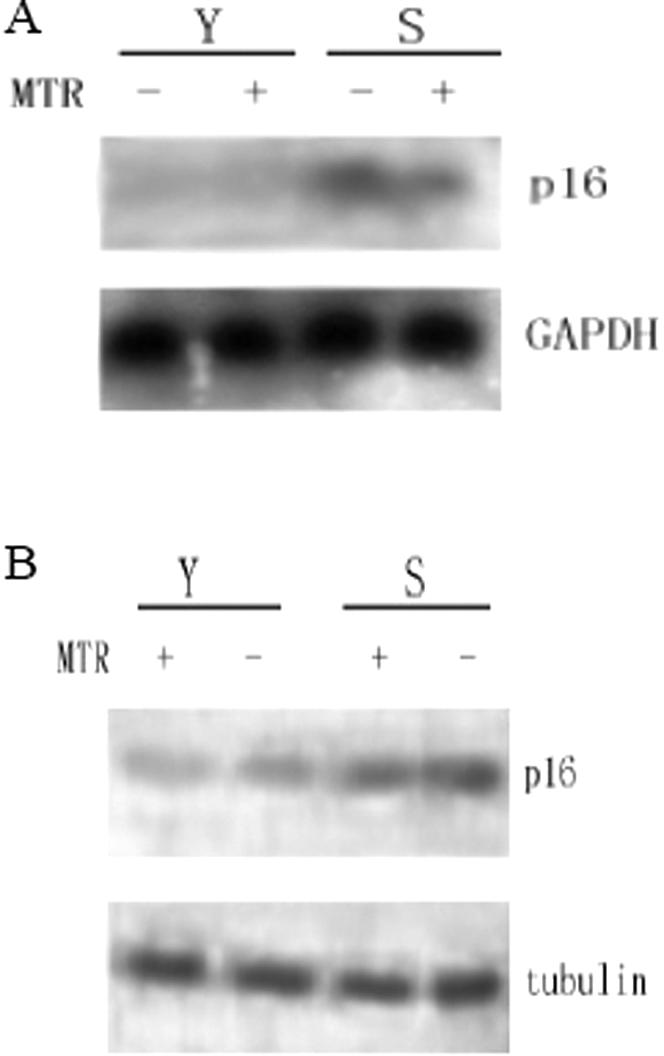
Expressions of *p16^INK4a^* mRNA and protein are inhibited by MTR treatment. Young (Y) and senescent (S) 2BS cells were either treated with 100 nM MTR (MTR) for 24 hr or left untreated, total RNA and protein were prepared and subjected to analyze the expression of indicated genes by Northern blotting (A) and Western blotting (B), respectively.

To confirm the effect of Sp1 on *p16^INK4a^* promoter activity, mithramycin (MTR), which specifically blocks Sp1 and Sp3 binding to GC boxes, was added to 2BS cells after transfection with pGL3-620. As expected, the *p16^INK4a^* promoter activity was inhibited by MTR in a dose-dependent manner in both young (Fig. 5A) and senescent (Fig. 5B) 2BS cells. Further, the *p16^INK4a^* expression was also reduced at mRNA (Fig. 6A) and protein (Fig. 6B) levels in 2BS cells, by 66% (mRNA level) and 48% (protein level) respectively, in the senescence group with 24 hours of MTR treatment.

**Figure 7:**
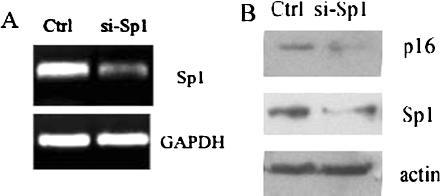
Knock-down of Sp1 reduces expression of endogenous *p16^INK4a^.* Following transfection of 2BS cells with si-Sp1 or a control plasmid, RT-PCR (A) and Western blotting (B) were carried out to analyze the expression of the genes indicated.

As the mechanism of MTR treatment is blocking the transcription factors such as Sp family binding to GC-box, in this way, MTR could also affect the binding of other transcriptional factors to *p16^INK4a^* promoter. To investigate the role of Sp1 specifically, Sp1 was knocked-down by RNAi to further confirm Sp1 binding to GC boxes is essential for the transcription of *p16^INK4a^* (Fig. 7A). Western Blot showed that si-Sp1 remarkably reduced the expression of Sp1, which in turn lead to a reduction of *p16^INK4a^* expression (Fig. 7B).

The results from two aspects mentioned above suggest that not only Sp1 can activate *p16^INK4a^* promoter in vivo but is required in maintenance of normal level of p16^INK4a^ protein in cultured human fibroblasts.

### The binding affinity to *p16^INK4a^* promoter is the key event but not the quantity of Sp1 enhanced during the aging process

**Figure 8:**
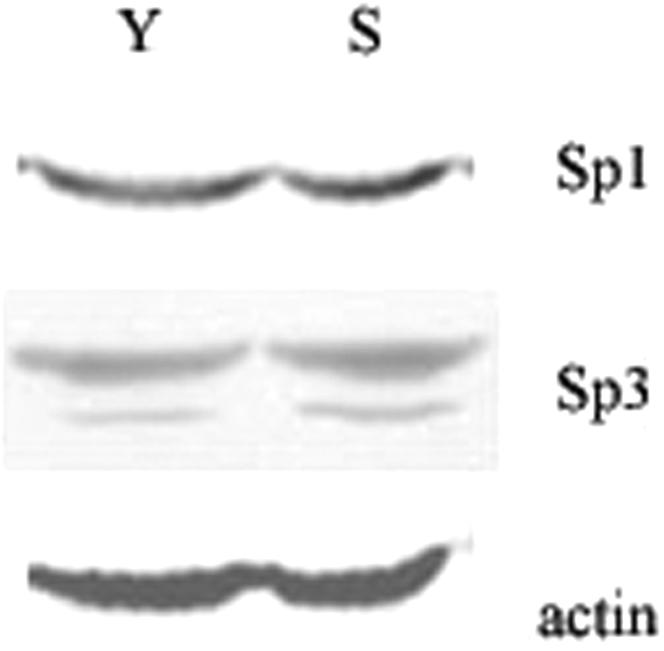
Expression of Sp1 and Sp3 in young and senescent 2BS cells. Western blot analysis of Sp1 and Sp3 expression in young (Y) and senescent (S) 2BS cells, data are representative of three independent experiments.

The results mentioned above demonstrate that during the cell aging process, Sp1 contributes to the higher expression of *p16^INK4a^.* However, which is responsible for this contribution: the quantity of the Sp1 expression or the binding affinity to *p16^INK4a^* promoter? To elucidate this confusing problem, western analyses were performed using nuclear proteins from young and senescent 2BS cells. The results showed that the quantity of Sp1 and Sp3 proteins does not increase in the senescent cells. In another words, it contains the same amount of Sp1 and Sp3 proteins as that of young cells (Fig. 8). Combined with the results observed in EMSA and ChIP assays, we thus conclude that the increased binding must be due to the increase of Sp1 DNA binding affinity, which in turn lead to the higher expression of *p16^INK4a^* in senescent cells.

### Sp1 increases SA-β-Gal activity whereas si-Sp1 expression inhibits SA-β-Gal activity

**Figure 9:**
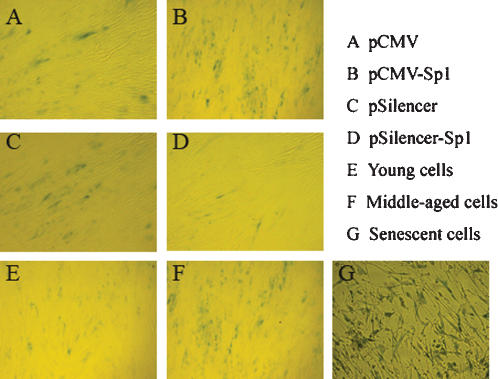
Influence of Sp1 levels on SA-β-Gal activity. 2BS/pCMV (A), 2BS/ pCMV-Sp1 (B), 2BS/pSliencer (C), 2BS/si-Sp1 (D), (all above at PD 40), untransfected young (PD 27) (E), middle-aged (PD 48) (F), and senescent (PD 56) (G) 2BS cells were cultured and then stained to assess SA-β-Gal activity.

SA-β–galactosidase staining is a common marker for cellular senescence. Usually, the β-galactosidase activity increases with the cell PD(population doubling) accumulating. The biological effect of over-expression (by Sp1 expression plasmid transfection) or knockdown (by RNAi approach) of Sp1 in 2BS cells were further evaluated by this method. The results showed that Sp1-overexpressed cells were strongly stained blue versus the control. However, there were only a few dispersed cells that were SA-β-Gal-stained in the Sp1 knocked-down cells (Fig. 9). All these outcomes manifested that RNAi-mediated silencing of Sp1 gene could delay senescence accompanied with decreased *p16^INK4a^* levels, however, over-expression of Sp1 could increase the *p16^INK4a^* expression and finally accelerate the onset of senescence of 2BS cells.

## Discussion

*p16^INK4a^*, a tumor suppressor gene that inhibits cyclin-dependent kinase 4 and cyclin-dependent kinase 6, has also been implicated in the mechanisms underlying replicative senescence. Many transcription factors are involved in transcriptional regulation via the corresponding elements distributed in the promoter region of *p16^INK4a^*. What should be emphasized is that the character of *p16^INK4a^* promoter is GC-rich rather than the common element such as TATA box. In addition, GC-rich boxes represent putative target sites for binding of Sp1 and Sp3 transcription factors [29]. In this study, we established the importance of transcription factor Sp1 on the regulation of *p16^INK4a^* gene expression from the different aspects and different strategies during aging in human embryonic lung fibroblasts, 2BS cells. Among the five GC boxes within the region of 620 bp upstream of the translation start site, the contribution of these five elements to *p16^INK4a^* transcription is not equal, GC-I, II and IV is more important rather than GC-III and V by mutagenesis analyses, especially GC-II is the most crucial element to the *p16^INK4a^* promoter activity, since its mutation abolished *p16^INK4a^* gene transcription in senescent cells. What's more, the effect on the promoter activity in senescent cells is more significant than in young cells. All results mentioned above suggested that GC boxes are more instrumental to *p16^INK4a^* gene expression coupled with aging process and it is the first time to investigate the roles of GC-boxes distributed in *p16^INK4a^* promoter and the relationship between these elements and transcription activity of the target gene meticulously and systemically during aging.

Protein binding studies identified Sp1 and Sp3 as the major components of the complexes formed between the nuclear extracts and the oligos containing GC box. Although we cannot preclude involvement of other factors, the Sp1 and Sp3 antibodies blocked each of the specific complexes in vivo and in vitro. Sp1 is a widely studied transcription factor that can bind to and act through the GC boxes. Although it is generally believed that Sp1 is ubiquitously expressed, Sp1 gene expression can show up to 100-fold differences in different cell types and at different stages of development in mouse [30]. While Sp3 is found to be highly homologous to Sp1 with similar affinities for GC and GT boxes, there are some striking functional differences between them. In some cell lines, it can activate transcription [31]. However, under other circumstances Sp3 is only weakly active, and in some cases, Sp3 can repress transcription driven by Sp1 or other transcription factors [32]. To investigate the transcription activities of Sp1 and/or Sp3 on *p16^INK4a^* promoter, we co-transfected Sp1 and/or Sp3 expression vectors with pGL3-620 into young and senescent 2BS cells and performed the luciferase assays. The results showed that Sp1 induced the *p16^INK4a^* promoter activity, while Sp3 had little effect. The induction of promoter activity was not as potent as we would expect, which might be due to the high levels of endogenous Sp1 expression and the lower transfection efficiencies of 2BS cells (The same experiments in HeLa cells showed the more evident change, data was not shown). To further prove the effect of Sp1 on *p16^INK4a^* expression, two strategies in different way were used: MTR, an inhibitor of Sp1/Sp3 binding, and siRNA, the inhibitor of Sp1 expression. The results which reduced the *p16^INK4a^* promoter activity and its expression both in mRNA level and protein level suggest again that Sp1 is both necessary and sufficient for the induction of *p16^INK4a^* gene expression in 2BS cells.

It has been documented that the expression of *p16^INK4a^* increased in senescent human fibroblast cells owing to the regulation of various of transcription factors in previous and our former work , such as E47, Id1, Jun B and RREB etc.[18], [20]–[22], [25]. In this study, both EMSA and ChIP assays showed that senescent cell nuclear extracts contained higher Sp1/Sp3 binding activities to the *p16^INK4a^* promoter. Taken together the results that mutation of GC boxes had more detrimental effect on the *p16^INK4a^* promoter in senescent cells and knock-down of Sp1 by siRNA down-regulate the expression of *p16^INK4a^*, we postulate that Sp1 transcriptional activity, including the binding activity to the GC boxes of *p16^INK4a^* promoter, is enhanced in senescent cells, which in turn plays a role in the elevated *p16^INK4a^* gene expression during senescence. It is applied a reasonable interpretation for the observation of much more potent promoter activities for *p16^INK4a^* in senescent cells.

In addition, though Sp1 is considered to be a constitutive transcription factor, Sp1 protein levels can vary significantly in different tissues. To test whether increased Sp1 binding is a result of induced Sp1 expression in senescent cells, we performed Western analyses. The results showed that the protein levels of Sp1 and Sp3 did not change significantly between young and senescent 2BS cells. So the increased binding observed in the senescent cells is likely the result of the augmentation of Sp1 and/or Sp3 binding affinity. On the other hand, the result of RNAi transfection demonstrated that although it need not increase Sp1 expression, however, a basal level is necessary for *p16^INK4a^* expression. Once it is lower than certain level, the expression of *p16^INK4a^* is also weakened.

The alteration of Sp1 activity during senescence may result from different post-translational modification or transcriptional co-factor. The two major types of modification that are thought to be involved in transcription regulation by Sp1 are glycosylation and phosphorylation. O-glycosylation of Sp1 with N-acetylglucosamine has been linked to multiple changes in Sp1 function, including altered self-association, altered interaction with basal transcription factors and modulation of its degradation [33], [34], but not its DNA binding activity [35], while phosphorylation has been found to either decrease [36] or increase [37] the DNA binding activity. Finally, earlier studies have shown that histone deacetylase 1 (HDAC1) binds to Sp1 and represses its transcription activity [38]. Sp1 may serve as a scaffold to recruit HDAC to the promoter, which causes chromatin condensation leading to transcription repression. Since the level of HDAC1 has been found to decrease significantly in senescent human fibroblasts [39], which may result in the release of Sp1, we postulate that more active Sp1 would be available for transcription activation.

All in all, among the multitudinous transcription factors, Sp1 is also an important and essential member for the transcription and expression of *p16^INK4a^*, whereas, in another aspect, GC box distributed in the promoter of *p16^INK4a^*, especially GC- II is also the key element for both the expression of *p16^INK4a^* and binding with Sp1. Only both of them interact properly, do the gene expression and cell physiology act in the normal program.
